# A reverse transcription-cross-priming amplification method with lateral flow dipstick assay for the rapid detection of *Bean pod mottle virus*

**DOI:** 10.1038/s41598-021-03562-8

**Published:** 2022-01-13

**Authors:** Qian-Qian Yang, Xing-Xing Zhao, Dao Wang, Peng-Jun Zhang, Xue-Nan Hu, Shuang Wei, Jing-Yuan Liu, Zi-Hong Ye, Xiao-Ping Yu

**Affiliations:** 1 Zhejiang Provincial Key Laboratory of Biometrology and Inspection and Quarantine, College of Life Sciences, China Jiliang University, Hangzhou, China; 2 Guangzhou Customs Technology Center, Guangzhou, China; 3 Shanghai Customs Technology Center, Shanghai, China

**Keywords:** Pathogens, Isolation, separation and purification

## Abstract

*Bean pod mottle virus* (BPMV) is a destructive virus that causes serious economic losses in many countries every year, highlighting the importance of its effective detection. In this study, we developed a fast reverse transcription-cross-priming amplification (RT-CPA) coupled with lateral flow dipstick (LFD) diagnostic method for BPMV detection. The RT-CPA-LFD assay that targets the coat protein gene of BPMV was highly specific against diagnosing four other common viruses transmitted by soybean seeds, i.e., *Southern bean mosaic virus* (SBMV), *Tomato ringspot virus* (ToRSV), *Arabis mosaic virus* (ArMV), and *Tobacco ringspot virus* (TRSV). The sensitivities of the real-time fluorescent RT-CPA and the RT-CPA-LFD assay were at least 50 pg/μl and 500 pg/μl, respectively. Despite a compromise in the limit of detection of the RT-CPA method compared with TaqMan-MGB real-time RT-PCR, our results demonstrated a notably better performance in the detection of field samples of BPMV-infested soybean seeds. With the advantages of efficiency and convenience by visual determination, the RT-CPA-LFD assay presents a potential application for the rapid and accurate detection of BPMV in routine tests.

## Introduction

*Bean pod mottle virus* (BPMV) is a comovirus that has a bipartite positive-strand RNA genome consisting of RNA-1 and RNA-2^1,2^. It was first described on the common bean *Phaseolus vulgaris* L. var. Tendergreen^3^ and was recognized to infest soybeans in the field in 1951^4^. BPMV has become one of the most economically important soybean viruses in the U.S. by causing the delay of maturation of soybeans with the symptoms of a green stem and seed coat mottling^5–7^. Infection of BPMV reduces seed yield with general losses of 3–52% depending on the variety, geographical range, infection time, etc.^2,8^. Coinfection of BPMV and *Soybean mosaic virus* (SMV) can reduce soybean yield up to 85%^9^. In addition, BPMV has secondary effects on soybean quality, including increasing *Phomopsis longicolla* seed infection^1^ and facilitating systemic movement of *Soybean vein necrosis virus* (SVNV)^5^.

In addition to the major soybean-growing areas of the southern and southeastern United States^2^, BPMV occurs in Canada^10^, Brazil^11^, Peru^12^, Ecuador^13^, and Iran^14^. Natural spread of BPMV was mainly through vectored leaf-feeding beetles in the field, but transportation of seeds by human has enabled its the long-distance spread^1^. For example, BPMV is frequently intercepted from imported soybeans at entry ports. China, which is one of the largest importers of soybeans, has listed BPMV among the plant quarantine pests due to its serous damage. Several methods for detecting BPMV have been developed, including serological and PCR-based methods, such as enzyme-linked immunosorbent assay (ELISA)^15^, reverse transcription polymerase chain reaction (RT-PCR)^16^, semi-nested RT-PCR^17^, and multiplex RT-PCR^18^.

Although PCR-based detection has been widely applied in many fields, it has been largely limited to point-of-care analysis due to the requirement of large and costly thermal cyclers^19^. Serving as promising alternatives to PCR, isothermal amplification technologies have developed rapidly since their immergence in the 1990s^19,20^. Due to the notable advantage of rapid and sensitive detection of DNA and RNA, isothermal amplification technologies have been widely applied in detecting microorganisms and are thus increasingly employed in diagnosing human diseases^21^, food pathogens^22^, and quarantine pests^23^. For example, isothermal amplification technologies have been established for more than 20 plant virus species that are officially listed as quarantine pests in the P. R. China.

The CPA isothermal DNA amplification technology was developed by Ustar Biotechnologies (Hangzhou, China). It relies on the application of a strand-displacing polymerase and it is characterized of high specify and sensitivity in amplification with five primers, namely one cross primer, two amplification primers, and two displacement primers^24^. CPA was initially designed for rapid diagnosis of tuberculosis by detecting the pathogenic bacteria *Mycobacterium tuberculosis*^25^. Recently, this technology has also been increasing employed for GMO screening^26^, detection of food pathogens^27^ and animal diseases^28^. For plant quarantine pests, CPA has been developed and shown high efficiency in detecting such as *Prunus necrotic ringspot virus* (PNRSV)^29^ and *Cucumber green mottle mosaic virus* (CGMMV)^30^.

Different methods have been developed for determining amplicons from isothermal nucleic acid amplification technologies, such as electrophoresis gels, fluorescent dyes, and lateral flow dipsticks (LFD). Among these methods, the LFD method, which based on lateral flow immunoassay can visually determine double-labeled amplicons within the short timeframe of 5–10 min^31^, has a great advantage in detecting amplicons. In this study, we aimed to establish an RT-CPA-LFD method combined with reverse transcription (RT) to amplify BPMV RNAs and evaluate the potential of this method for detecting BPMV in soybeans.

## Materials and methods

### Samples, total RNA extraction, and cDNA synthesis

In addition to BPMV detection, we also included SBMV, ToRSV, TRSV, and ArMV to assess the specificity of the CPA-LFD method. All inactivated virus samples were obtained from the Chinese Academy of Inspection and Quarantine (Beijing, China) and were individually propagated in White Burley *Nicotiana tabacum* cv. and other host plants. Soybean seeds imported from the USA, which had suspected BPMV infection with seed coat mottling or bleeding hilum symptoms that were captured by Guangzhou Customs District of China, were used as field samples. Healthy soybean seeds purchased from a local market in Hangzhou, China, were used as a negative control. Distilled RNase-free water was used as a blank control. All samples were kept in a sealed container with anhydrous calcium chloride as the desiccant at − 80 °C. This study complies with relevant institutional, national, and international guidelines.

Total RNA was extracted by using the MiniBEST Universal RNA Extraction Kit (TaKaRa, Japan) following the manufacturer’s protocols from 50 mg of White Burley leaves or whole soybean seeds. The quality and concentration of the extracted RNA were estimated with an ND2000 spectrophotometer (NanoDrop Technologies). cDNA was synthesized using a PrimeScript™ II 1st Strand cDNA Synthesis Kit (TaKaRa, Japan) using the primers Random 6 mers and Oligo dT Primer provided in the kit.

### Amplifying and sequencing the coat protein gene of BPMV

Based on the analysis of the genomic sequence of BPMV (GenBank accession number GQ996950), we designed primers for amplifying a portion of the coat protein (CP) gene. The primers were BPF (5-CCAATCCTGGTTTGAAGAATC-3) and BPR2 (5-ACGAGAGGGTCATGCCTAATTTC-3) to amplify ~ 1900 bp fragments. PCR was performed in 50 µl reactions containing 25 µl of 2 × TransStart FastPfu PCR Super Mix, 0.5 µM each of the primers, 100 ng of cDNA, and RNase Free ddH_2_O. The PCR conditions were 94 °C for 3 min, 34 cycles of 98 °C for 10 s, 59 °C for 30 s, and 72 °C for 120 s, and a final elongation at 72 °C for 8 min. The amplicons were purified using the SanPrep Column PCR Product Purification Kit (Sangon Biotech, Shanghai, China) and then cloned with the pMD19-T Vector Cloning Kit (TaKaRa, Japan) after adding poly A tail to the purified PCR fragments. The recombinant bacmids were then transformed into T-Fast competent *E. coli* (Tiangen, China), screened by blue-white selection, and sequenced by the M13 primer at Sangon Biotech (Shanghai). The sequences were examined for errors with the program Geneious 11.1.5 and verified with BLASTn in GenBank after manual editing.

### CPA primer design and synthesis

Five sets of CPA primers (S1–S5) were designed according to the CP gene sequence using Geneious 11.1.5. Each set of primers included a pair of peripheral displacement primers (BPBF/BPBR), a pair of amplification primers (BPDR/BPMBR), and a cross primer (BPCPF) (Table 1). Primers BPBF/BPBR and BPDR/BPMBR were designed with following the criteria: length 18–20 bp, GC content 40–50%, differences in Tm values of each pair within 5, and amplicon sizes of 150–200 bp. The cross primer was 40 bp with a 5′-end sequence identical to that of BPMBR, followed by a GT sequence for connection, and the rest of the section was complimentary to the target sequences. Detailed information on the five primer sets is shown in Supplementary Table S1. The primers were synthesized by Sunny Biotechnology (Shanghai, China).

**Table 1:** The primer set S1 screened with high efficiency in the RT-CPA-LFD assay for BPMV detection.

Name	Sequences (5′–3′)	Length (bp)	GC%
BPBF	CACAGTTGGAGGAACCATT	19	47.4
BPCPF	CTTTAATACGATGAGTGCGGTGCGTAGTGATCTATTGGCA	40	45.0
BPDR	FAM-GCAAATCAACACTTGCTCT	19	42.1
BPMBR	Bio-CTTTAATACGATGAGTGCG	19	42.1
BPBR	TAGCACAGGCCAATGCTAT	19	47.4

### Primer evaluation using real-time fluorescent RT-CPA

Real-time fluorescent reverse transcription-CPA (RT-CPA) was carried out to detect the efficiency and specificity of the primers using a Bio-Rad CFX Connect Real-Time System (America). The total RNA extracted from the healthy leaves of White Burley was used as a negative control. A real-time fluorescent RT-CPA reaction was performed in a total volume of 20 µl containing 8 U of *Bst* DNA polymerase, 10 U of AMV reverse transcription polymerase, 0.3X ThermoPol buffer, 12.5 mM betaine, 2 mM MgSO_4_, 0.1 mM dNTP, 2.5 µM SYTO™ 16 Green Fluorescent Nucleic Acid Stain (Thermo Fisher), 1 µM BPCPF, 0.6 µM BPDR, 0.6 µM BPMBR, 0.2 µM BPBF, 0.2 µM BPBR, and 50 ng RNA template. Reactions were carried out with a Bio-Rad CFX Connect Real-Time System (America) at isothermal temperature (60 °C) for 90 min. FAM was chosen as the selector fluorescent light channel with an excitation wavelength of 495 nm and an emission wavelength of 517 nm.

Specificity tests were performed by applying the real-time fluorescent RT-CPA assay to the total RNA extracted from the leaves of White Burley infected by BPMV, SBMV, ToRSV, ArMV, and TRSV.

### Development of the RT-CPA-LFD assay for BPMV detection

The best set of primers selected for the real-time fluorescent RT-CPA assay was modified to develop the primers used for RT-CPA-LFD. The 5′-ends of the amplification primers BPDR and MBR were labeled with 6-FAM and biotin, respectively (Table 1). The RT-CPA-LFD assay was performed on a BIO-RAD T100TM Thermal Cycler (America) at 60 °C for 90 min (1 min per cycle) using the same protocol as the real-time fluorescent RT-CPA assay except in the absence of SYTO™ 16 Green Fluorescent Nucleic Acid Stain. The lateral flow dipstick (LFD) of Tiosbio^®^ LF-detect (Beijing Baoying Tonghui Biotechnology Co., Ltd.) with fluorescein isothiocyanate (FITC) and biotin was used to detect the RT-CPA amplicons. The sample immersion area of the Tiosbio^®^ LF-detection dipsticks was inserted directly into the RT-CPA solution without dilution and incubated for 2 min in an upright position, and the test results were immediately interpreted visually. When the test and control bands were clearly formed as red and blue lines, respectively, the test was considered positive, whereas when only the control band was visible as a blue line, the result of the test was negative.

### Specificity test of the RT-CPA-LFD assay for BPMV detection

We then tested the specificity of the developed RT-CPA-LFD assay to detect BPMV by utilizing the total RNA extracted from the leaves of White Burley infected by BPMV, SBMV, ToRSV, ArMV, and TRSV. All RNA templates were tested at a uniform concentration of 50 ng/µl.

### Sensitivity of the real-time fluorescent RT-CPA and RT-CPA-LFD assays

Seven gradient concentrations of RNA templates with BPMV were prepared by tenfold serial dilutions of the initial 50 ng/µl solution in RNase-free ddH_2_O. The sensitivities of the real-time fluorescent RT-CPA and the RT-CPA-LFD assays were tested by applying the gradient concentrations of RNA templates with BPMV. Moreover, TaqMan-MGB real-time RT-PCR was employed to compare the detection sensitivities with the real-time fluorescent RT-CPA and RT-CPA-LFD assays established in this study.

The primers (BPMV-F/BPMV-R) and probe (BPMV-P) for detecting BPMV by real-time RT-PCR were published by Liu et al.^32^. TaqMan-MGB real-time RT-PCR was performed in 25 µl reactions containing 1 × FastKing One Step Probe RT-qPCR MasterMix (Tiangen, China), 1 × FastKing Enzyme Mix, 0.25 µM BPMV-F, 0.25 µM BPMV-R, 0.25 µM BPMV-P, and 50 ng RNA template. The conditions were 50 °C for 30 min and 95 °C for 3 min, followed by 40 cycles of 95 °C for 15 s and 60 °C for 30 s.

### Validation of the real-time fluorescent RT-CPA and RT-CPA-LFD assays

We validated the established RT-CPA-LFD assay for BPMV detection by examining on-field soybean seed samples with a recombined plasmid of a BPMV fragment as a positive control and a healthy soybean seed as a negative control, as well as comparing the real-time fluorescent RT-CPA and TaqMan-MGB real-time RT-PCR methods.

## Results

### Primer screening based on real-time fluorescent RT-CPA for BPMV detection

**Figure 1:**
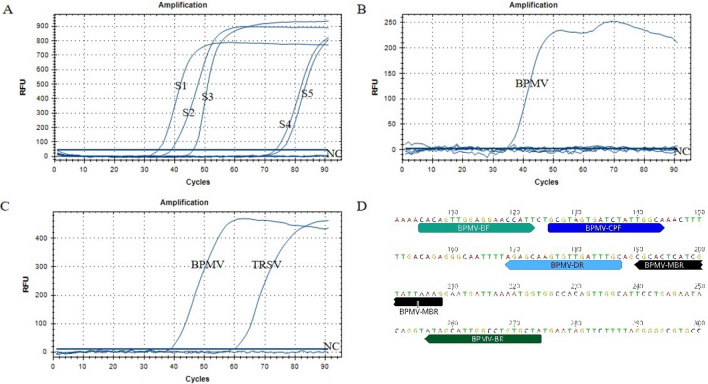
Primer set screening by real-time fluorescent RT-CPA for BPMV detection. (**A**) Reaction curves of the five sets of primers S1–S5. (**B**–**C**) Specific tests for primer sets S1 and S2 on five common plant quarantine viruses infesting soybean seeds: SBMV, ToRSV, ArMV, TRSV, and BPMV. (**D**) Primer location of primer set S1 on the BPMV coat protein. Arrows show directions. One cycle represents 1 min in real-time fluorescent RT-CPA. NC, negative control with RNase-free distilled water.

Using template RNA extracted from BPMV-infested White Burley leaves, five sets of primers (S1–S5, Table 1, Supplementary Table S1) were tested for their reaction efficiency in detecting BPMV by real-time fluorescent RT-CPA. Reactions with primer set S1 showed fluorescence signals the fastest, which reached their strongest signal at ~ 50 min, followed by reactions with primer set S2 (Fig. 1A). The signal was > 10% stronger at the plateau phase when S2 was used instead of S1. Although the reactions with primer set S3 showed similar fluorescent signals at the plateau phase as those of S2, the reactions started slower. Fluorescent signals were detected from reactions with primer sets S4 and S5 after 70 min and reached their highest at times longer than 90 min. Therefore, we chose primers S1 and S2 for further specificity evaluation tests.

By using the total RNA extracted from plant leaves infested with BPMV, SBMV, ToRSV, ArMV, and TRSV, primer S1 showed good specificity by working only on RNA from BPMV (Fig. 1B). However, the reaction with primer set S2 showed amplification curves from TRSV in addition to BPMV, indicating poor specificity (Fig. 1C). Primer set S1 (Fig. 1D) demonstrated the best efficiency among the five primer sets and was selected to perform the RT-CPA-LFD assay.

### Development of the RT-CPA-LFD assay for BPMV detection and the specificity test

**Figure 2:**
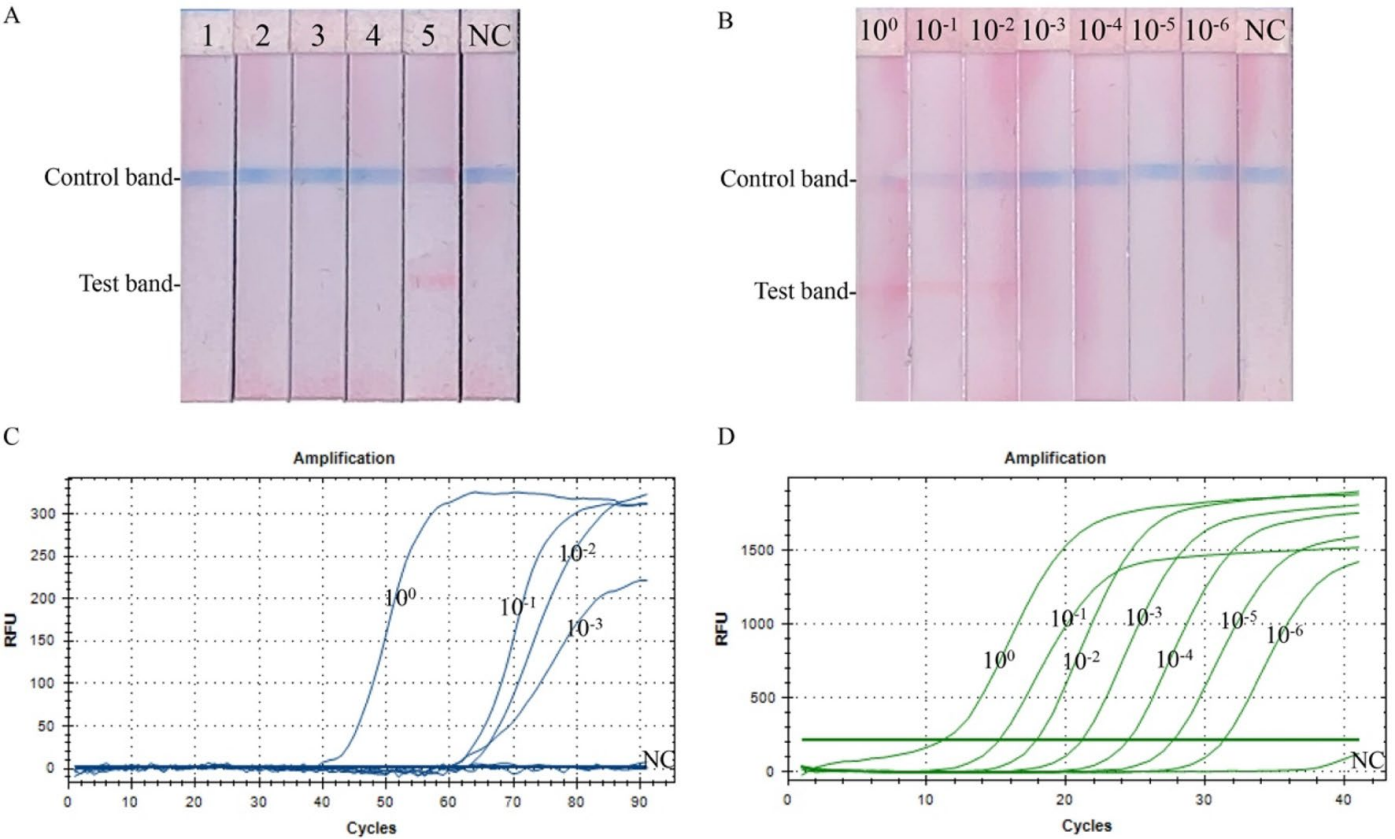
Evaluation of BPMV detection by the RT-CPA-LFD assay. (**A**) Specificity of the RT-CPA-LFD assay for BPMV detection. 1–5, RNA templates of leaves infested with SBMV, ToRSV, ArMV, TRSV, and BPMV, respectively. (**B**–**D**), Sensitivity test for BPMV detection by RT-CPA-LFD, real-time fluorescent RT-CPA, and TaqMan-MGB real-time RT-PCR. Dilutions (10^0^–10^−6^) of total RNA of the BPMV-infected leaf sample were with the initial concentration of 50 ng/µl. NC, negative control with RNase-free distilled water.

With the selected primer set S1, RT-CPA was performed on total RNA of the BPMV-infected White Burley leaves for 90 min, following visualization of the resulting amplicons with LFD strips. The test and control lines turned red and blue, respectively, within two minutes (Fig. 2A, lane 5). In contrast, the negative reactions with RNase-free distilled water showed only the blue control line (Fig. 2A, lane NC).

Total RNA extracted from White Burley leaves infested with SBMV, ToRSV, ArMV, and TRSV was used to assess the specificity of RT-CPA-LFD for BPMV detection. All of the RT-CPA reactions on the four viruses showed blue lines at the control line positions, but none of them showed red lines at the test line positions (Fig. 2A, lanes 1–4).

### Sensitivity evaluation of the RT-CPA-LFD assay for BPMV detection

Using serial tenfold dilutions of total RNA (50 ng/μl) prepared from BPMV-infected White Burley leaves as templates, we evaluated the sensitivity of RT-CPA-LFD to BPMV detection. Moreover, the sensitivity of RT-CPA-LFD was compared to the real-time fluorescent RT-CPA and TaqMan-MGB real-time RT-PCR methods. RT-CPA-LFD was able to detect the 10^0^, 10^−1^, and 10^−2^ diluted RNAs showing colors at both the control and test lines with the color of the test lines lighter as decreasing the concentration (Fig. 2B). No test line was detected in the samples containing 10^−3^ diluted RNAs and below (Fig. 2B).

Fluorescence signals were not observed at concentrations lower than 10^−3^ diluted RNAs in real-time fluorescent RT-CPA (Fig. 2C), while TaqMan-MGB real-time RT-PCR detected all seven dilutions of RNA templates with amplification curves (Fig. 2D). These results indicated that TaqMan-MGB real-time RT-PCR was at least 10,000 times higher sensitive than the RT-CPA-LFD assay in detecting BPMV.

### RT-CPA-LFD assay practice for BPMV detection on field samples

**Figure 3:**
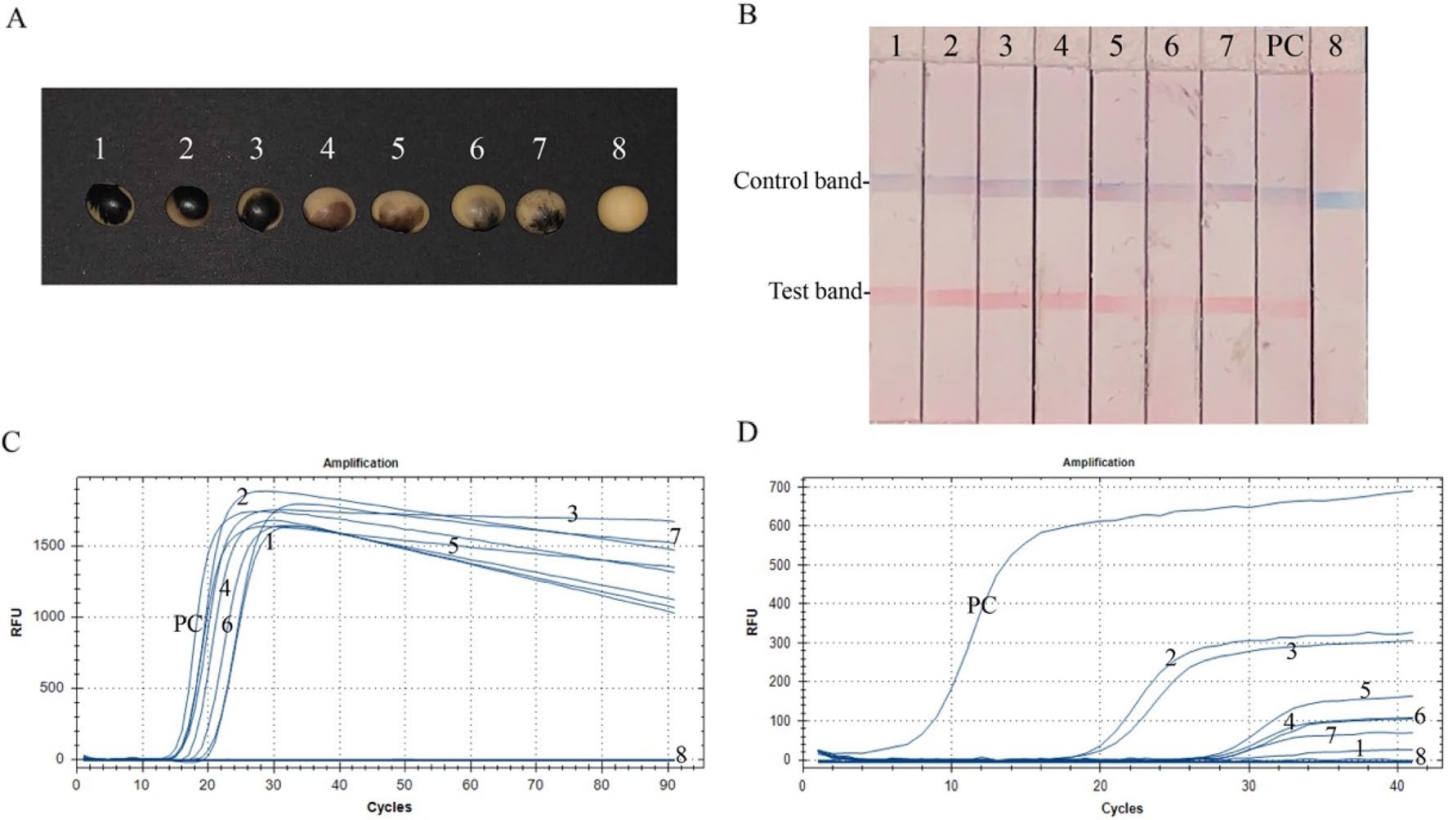
Validation of the BPMV detection methods on field samples. (**A**) Soybean seeds. 1–7, imported soybean seeds with suspected symptoms of BPMV infection; 8, healthy soybean seed from a local supermarket. (**B**–**D**) Detection of BPMV by RT-CPA-LFD, real-time fluorescent RT-CPA, and TaqMan-MGB real-time RT-PCR. PC, positive control of the RNA template from the BPMV-infested leaves. NC, negative control (a healthy soybean seed).

The RNA concentrations of the soybeans ranged from 27.5 to 509.4 ng/μl (Fig. 3A, Supplementary Table S2). Reactions with all seven field soybean samples turned red at the test line position and blue at the control line position, which was the same as that of the positive control (Fig. 3B). Amplification curves of the seven field soybean samples and the positive control were observed with amplification curves by real-time fluorescent RT-CPA, with the fluorescent signals appearing at 13–20 min and reaching their highest signal at approximately 30 min (Fig. 3C). TaqMan-MGB real-time RT-PCR showed the strongest signal for the positive control; however, all the field soybean samples demonstrated much weaker signals that were observed after longer times (Fig. 3D). The two higher concentration samples (seed number 2 and 3) performed better in the TaqMan-MGB real-time RT-PCR detection assay (Fig. 3D). All three methods produced negative results for the healthy soybean samples.

## Discussion

The isothermal amplification technology CPA has been shown to be effective in the detection of human, animal, and plant pathogens^25,29,33^. In this study, we designed primers and established a RT-CPA-LFD method targeting the coat protein of BPMV, which is an important quarantine soybean virus with RNA genomics. The CPA reaction was combined with reverse transcription to amplify the RNAs of BPMV at a constant temperature of 60 °C and could finish in no more than 90 min with the prepared template. The produced amplicons were subsequently visually distinguished within 2 min when coupled with lateral flow dipsticks, which enabled the assays to be easier and more useful. Our RT-CPA-LFD method for detecting BPMV is highly specific and has proven efficiency for detecting BPMV-infected field samples. The RT-CPA-LFD assay has demonstrated good potential for the field surveillance of BPMV.

The sensitivity of RT-CPA-LFD were at least 500 pg/μl, while that of the real-time fluorescent RT-CPA was tenfold higher. PCR is normally the gold standard for diagnosing plant diseases due to its superior sensitivity^34,35^. In particular, TaqMan-MGB real-time RT-PCR has been established as a national standard for many plant quarantine pests. In this study, TaqMan-MGB real-time RT-PCR for BPMV detection showed much higher sensitivity with lower concentration limits than the CPA methods for BPMV detection. However, the CPA methods performed much better than TaqMan-MGB real-time RT-PCR in detecting field soybean samples. One of the main reasons for this result is that PCR-based methods require much higher-quality nucleic acid solutions than isothermal amplification technologies^36–38^. Because plant tissues vary in the content of starch, protein, oil, polysaccharides, phenolic, etc., there has been no universal procedure for extracting good-quality RNA for all plant tissues^39,40^. The real-time quantitative PCR is often restricted by the low-quality RNA which contains the above-mentioned plant substance acting as inhibitors. Seeds, as complex storage organs, have higher challenge for extracting high-quality RNA than many other tissues of plants^40^. Thus, qPCR often fails when using the RNA extracted from seeds using the commercial kits^41,42^. qPCR-based methods usually require templates been purified when detecting plant pathogenic bacteria or viruses^43^. In contrast, isothermal amplification technologies are more tolerant to the inhibitory contaminants in RNA/DNA extraction. Isothermal amplification technologies have demonstrated notable advantages in effectively amplifying DNA/RNA targets from crude plant extracts. For example, RT-LAMP was 100 times more sensitive than RT-PCR in detecting crude extracts of *Tomato spotted wilt virus* (TSWV)^44^. RPA was proven effective in detecting crude extracts from *Tomato yellow leaf curl virus* (TYLCV)-infected tomato and bean leaves and *Banana bunchy top virus* (BBTV)-infected banana leaves with high sensitivity; however, PCR-based detection was not able operate on these crude extracts as templates^45,46^. Our results indicate that CPA also has the potential for application to extracts of low quality or crude extracts for plant disease diagnosis.

As a destructive virus that causes very large economic losses in many countries every year, the effective detection of BPMV has high importance. In addition to conventional immunological and PCR-based methods, reverse transcription loop-mediated isothermal amplification (RT-LAMP) was developed to detect BPMV, which is effective in the detection of BPMV-infected soybean seeds containing the fluorescent dye SYBR GREEN I^47^. With the similarity in displacement primers releasing the newly synthesized complementary chain, i.e., the peripheral displacement primers in the 4 s/5a region for CPA^24,25^ and the outer primers F3/B3 for LAMP^48,49^, both methods displayed the similar advantage of high sensitivity and disadvantage of false positives. For example, a comparison between the LAMP and CPA has shown that both methods are implemented in preliminary African swine fever (ASF) diagnosis with CPA having higher sensitivity^28^. Similar to LAMP, which had a high false positive rate caused by carryover contamination free in the air^34^, our results also proved a high false positive rate of CPA in detecting BPMV upon recognition of the amplification products using electrophoresis (data not shown). Therefore, methods that avoid opening tubes by adding color indicators to the reactions or access to the LFD in a sealed device were employed to detect the amplified products to resolve this problem.

A disposable nucleic acid detection device, a nucleic acid test strip cassette (NATSC) packed with an immunochromatographic detection strip in a sealed cartridge, is commercially available from Ustar (Hangzhou, China). RT-CPA coupled with this test strip cassette for vertical flow visualization detection of plant quarantine pathogens, such as *Prunus necrotic ringspot virus* (PNRSV)^29^, *Cucumber green mottle mosaic virus* (CGMMV)^30^, genetically modified organisms (GMO)^26^ and *Pseudomonas syringae* pv. *lachrymans*^50^ were developed. By establishing the RT-CPA-LFD method for BPMV detection, we further confirmed the reliability of CPA as a rapid detection method for routine tests of various plant pathogens. This combination of CPA and LFD technology is intended to be applied for plant disease diagnosis in point-of-care testing.

The isothermal amplification technologies have facilized the fast diagnosis of nuclear acid. Despite we performed the RT-CPA-LFD assay in a thermal cycler, the amplification device can be replaced by a simple single-temperature heat (e.g., water bath, metal bath, or thermal chemical units). The key step hampers an in-field application of our RT-CPA-LFD assay is lacking of simple and instrument-free techniques for extracting nuclear acid. During the last decade, researchers have made great efforts to simplify the nucleic acid isolation process from plant tissues. A couple of new techniques and devices have been developed for on-site DNA extraction from plant samples^51^. However, the techniques for on-site RNA extraction from plants were still very rare. The simplified methods for RNA extraction from human pathogens have forged ahead to speed the “sample-in-result-out” disease detection^51^, which may shed light to the development of similar methods for RNA extraction from plants in the future.

In conclusion, RT-CPA-LFD is an efficient method for detecting BPMV from infested plant leaves and soybean seeds. With its advantages of high specificity as well as visualization, this method has potential application in the routine fast on-site diagnosis of BPMV in plant inspection and quarantine.

## Supplementary Information

Supplementary Tables.
