# Supplementary material for: A reverse transcription-cross-priming amplification method with lateral flow dipstick assay for the rapid detection of Bean pod mottle virus
Source: Sci Rep. 2022 Jan 13;12:681. doi: 10.1038/s41598-021-03562-8 (PMC8758742; doi:10.1038/s41598-021-03562-8)
Supplement: Supplementary file 1 — Supplementary Tables. [file 41598_2021_3562_MOESM1_ESM.docx]

**Supplementary Materials**

**Table S1** Other primer sets used for evaluating the RT-CPA-LFD assay for BPMV detection.

| Primer sets | Primer name | Sequences (5’-3’) | Length (bp) | GC% |
| --- | --- | --- | --- | --- |
| S2 | BPBF | ACACCATCTGCTCTCAAGA | 19 | 47.4 |
|  | BPCPF | GAAATTCCAAACTCCATGCCAGTGAATTATGGAATCCTGC | 40 | 42.5 |
|  | BPDR | CAGAACACGGGTTTGGATT | 19 | 47.4 |
|  | BPMBR | GAAATTCCAAACTCCATGC | 19 | 42.1 |
|  | BPBR | CCATCCAGTGACACAAATG | 19 | 47.4 |
| S3 | BPBF | TGCAGGATGTTCAGGTTAC | 19 | 47.4 |
|  | BPCPF | TGCAGGATGTTCAGGTTAC | 40 | 45 |
|  | BPDR | CCATCCACCTATTTAACAC | 19 | 42.1 |
|  | BPMBR | TTAACACTTCGGGAAGTGC | 19 | 47.4 |
|  | BPBR | CCTATAGAAAGAGGCATTC | 19 | 42.1 |
| S4 | BPBF | GTTGGAGATCTCGTCTTTG | 19 | 47.4 |
|  | BPCPF | AATCTCTCCAAACTGCACCCTCTAAGATGACTTCTCCCTA | 40 | 45.0 |
|  | BPDR | ATTGATGGTGTCATCAGCC | 19 | 47.4 |
|  | BPMBR | AATCTCTCCAAACTGCACC | 19 | 47.4 |
|  | BPBR | GAGTTGACCAAGCTGTAAG | 19 | 47.4 |
| S5 | BPBF | CCTGCAACAACTCTGTTGG | 19 | 52.6 |
|  | BPCPF | ATTGAGCCCATATGCACACGTGACGGGTGTCCATATTTGT | 40 | 47.5 |
|  | BPDR | CACCTGGTATTGTAGACAC | 19 | 47.4 |
|  | BPMBR | ATTGAGCCCATATGCACAC | 19 | 47.4 |
|  | BPBR | CCAGAAGACGAGAACCTGA | 19 | 52.6 |

**Table S2** RNAs used for application tests of the RT-CPA-LFD assay for BPMV detection. 1-7, field soybean seeds; NC, negative control of a healthy soybean seed; 8, positive control of a BPMV-infected White Burley leaf.

| Sample code | Concentration (ng/µl) | OD value (260/280) |
| --- | --- | --- |
| 1 | 72.6 | 2.11 |
| 2 | 509.4 | 2.11 |
| 3 | 416.5 | 2.15 |
| 4 | 27.5 | 2.26 |
| 5 | 162.9 | 2.18 |
| 6 | 106.1 | 2.17 |
| 7 | 136.7 | 2.19 |
| 8 | 500 | 2.09 |
| NC | 65.7 | 2.04 |
